# Practice What We Preach: Beginning a Journey to Embrace Patient-Centered Outcomes Research

**DOI:** 10.3390/nursrep11030068

**Published:** 2021-09-17

**Authors:** Huey-Ming Tzeng, Bridget E. Hawkins, Anne Howard, Sharon Woodfox-Ryan, Aisen Chacin, Maribel M. Marquez-Bhojani, Kenneth M. Johnson, Michelle Sierpina, James Grant, Deborah J. Jones, Lorraine S. Evangelista

**Affiliations:** 1 School of Nursing, The University of Texas Medical Branch at Galveston, Galveston, TX 77555, USA; debjjone@utmb.edu (D.J.J.); lsevange@utmb.edu (L.S.E.); 2 RISE Center, School of Nursing, The University of Texas Medical Branch at Galveston, Galveston, TX 77555, USA; behawkin@utmb.edu; 3 Moody Medical Library, The University of Texas Medical Branch at Galveston, Galveston, TX 77555, USA; anhoward@utmb.edu; 4 African American Faith Community Nurse, International Nurses Unit, Church of God in Christ, Inc., Texas City, TX 77590, USA; swryan2@gmail.com; 5 Medical Fabrication Lab, MakerHealth, Department of Nursing Science Innovation, Health System, The University of Texas Medical Branch at Galveston, Galveston, TX 77555, USA; acchacin@utmb.edu; 6 School of Nursing, University of St. Thomas, Houston, TX 77006, USA; mmbhojan05@yahoo.com; 7 Department of Pharmacology and Toxicology, School of Medicine, The University of Texas Medical Branch at Galveston, Galveston, TX 77555, USA; kmjohnso@utmb.edu; 8 Osher Lifelong Learning Institute, The University of Texas Medical Branch at Galveston, Galveston, TX 77555, USA; msierpin@utmb.edu; 9 Galveston Baptist Association, Dickinson, TX 77539, USA; director@galvestonbaptist.org

**Keywords:** community engagement, patient engagement, patient-centered outcomes research, fall prevention

## Abstract

Background: Patient-centered outcomes research seeks to answer patient-centered questions. The process includes varied locations and individuals throughout the care continuum to address individual differences and constraints in implementation and dissemination. Problem: This paper intends to answer this question: do academic nurses practice what they preach by assisting patient-centered outcomes research and researchers through their engagement with patients, caregivers, and other community stakeholder partners in nursing research? Approach: This paper provides an overview of how academic nurses in a single institution (the University of Texas Medical Branch at Galveston School of Nursing) began to embrace patient-centered outcomes research. Conclusion: Whether academic nurses are practicing what they preach in terms of patient-centered outcomes research remains uncertain. More examples from academia are required to make that determination. Academic nurses worldwide have embarked on a steep learning curve to embrace patient-centered outcomes research. This journey will require patience and a systematic strategy.

## 1. Background

Adopting patient-centered outcomes research [1] by actively engaging patients, caregivers, and community organization partners is highly valued and recommended by funding agencies like the Patient-Centered Outcomes Research Institute. Patient-centered outcomes research, also known as community engagement or community involvement research, aims to make the voices of patients and their caregivers heard when assessing the value of their healthcare options and assisting them in communicating and making informed healthcare decisions [1,2]. Patient-centered outcomes research can include the patients, caregivers, and other stakeholder partners (for example, clinicians, policymakers), as appropriate. Patient-centered outcomes research can occur in diverse settings and include participants from across the care continuum (e.g., health care providers from primary care, secondary or tertiary settings). [1] One example is to identify the best way for health care providers to overcome obstacles to providing fall prevention education to community-dwelling older patients when they return home from acute hospitalization [3].

To improve nursing-care quality and patient safety, it is essential to engage and collaborate with the prospective end-users throughout all research stages. In addition, academic partners such as researchers and others in a school of nursing (hence referred to as academic nurses in this paper) may benefit from a better understanding of an issue that is under investigation through community engagement (for example, health equity) [2,4]. As a result, the purpose of this paper is to answer the question: do academic nurses practice what they preach by assisting patient-centered outcomes research and researchers through their engagement with patients, caregivers, and other community stakeholder partners in nursing research?

One recent study described the development of an infrastructure to cultivate a sustainable, community-academic research partnership with Meharry Medical College, a historically black college and university [5]. However, to the best of our knowledge, no literature has documented how patient-centered outcomes research has been integrated by academic nurses into any academic activity involving a research infrastructure to teaching patient-centered outcomes research skills. 

As for the purpose and focus of this paper, this paper provides an overview of how academic nurses at a single institution, the University of Texas Medical Branch at Galveston (UTMB) School of Nursing, began its journey to embrace patient-centered outcomes research and their progress so far. We used the strategic plan of the UTMB School of Nursing to demonstrate a systematic approach to embracing patient-centered outcomes research and supporting a researcher’s journey to fulfill her patient-centered outcomes research goal. In addition, we highlighted the process of developing the community-engagement infrastructure at the UTMB School of Nursing. We also determined that there is still much to be done to implement and support patient-centered outcomes research at this school of nursing. Several practical implications are discussed. The problem addressed in this paper focuses on overcoming the challenges to embrace patient-centered outcomes research in academic nursing settings.

## 2. Approach

Table 1 summarizes the related activities by dates at the UTMB School of Nursing. The UTMB School of Nursing began implementing a systematic approach to support patient-centered outcomes research in 2019. This strategic plan, titled “Vision 2025,” emphasized the importance of engagement by expanding professional networks and community interactions to generate opportunities for students, faculty, and alumni to promote long-term beneficial collaborations [6]. Population health was identified as one of six research areas of excellence by the UTMB School of Nursing’s Research Innovation and Scientific Excellence (RISE) Center [7], where community engagement and interdisciplinary collaborations would be used to investigate the strengths and needs of local and global populations to improve health and preventive care. The goal of the RISE Center is to facilitate interprofessional research collaborations and partnerships within the faculty from the School of Nursing, as well as other departments, universities, and community stakeholders. One of the RISE Center’s performance metrics is the number of community, national, and international connections and collaborations that have been established since its inception. 

The UTMB School of Nursing’s systematic approach is essential in the initial stages of research to support a researcher’s patient-centered outcomes research journey. The first author joined the UTMB School of Nursing in May 2019 and started a patient-centered outcomes research program based on previous experience. Here, we highlight a few patient-centered outcomes research activities within the UTMB School of Nursing’s RISE Center. We also include several testimonials from the patients, caregivers, and other community stakeholders who participated in or supported these activities.

### 2.1. Supporting a Researcher’s Patient-Centered Outcomes Research Journey

The first author’s vision to embrace holistic health through research on aging and patient-centered outcomes research was facilitated by receipt of the University of Texas System Rising Science and Technology Acquisition and Retention (STARs) Award of $250,000 for equipment, repair, and renovations funded from Permanent University Fund bond proceeds in August 2019. The first author is the primary recipient of this award. These funds were used to renovate a clinical laboratory space to be used for a new Community Space for Health and Well-Being (henceforth referred to as the Community Space) within the UTMB School of Nursing’s RISE Center. The refurbishment of this facility was completed in January 2021. The funds were also used to outfit a mobile classroom with items such as iPads (the 7th generation/version) (Apple, Inc., Cupertino, CA 95014, USA) and low- and high-technology home gadgets to deliver onsite activities that could be used to bring Community Space activities to the communities it serves (for example, UTMB’s branch of the Osher Lifelong Learning Institute [OLLI] [8] and Sealy Center on Aging’s Learning Center [9]). The Community Space is dedicated to aiding patient-centered outcomes research to improve the health and well-being of community-dwelling older people and their family caregivers.

The Community Space Design Advisory Committee, consisting of three patient and caregiver partners and four UTMB clinicians or other stakeholder partners (faculty from UTMB’s Health System Department of Nursing Science Innovation, MakerHealth Space’s [10] engineer, UTMB OLLI’s founding director, a UTMB medical reference librarian, and a registered nurse investigator), was formed in late 2019 and is still in operation. The first stage in developing a strategic plan for the Community Space was to create a mission, vision, and values statement. The preliminary versions of the mission, vision, and value statements were developed on the basis of feedback from the Community Space Design Advisory Committee beginning in the fall of 2020 through meetings, surveys, and interviews with five patients or caregivers.

This strategic planning process was carried out at no additional expense to the lead faculty or UTMB School of Nursing by a senor strategic planning analyst from the UTMB President’s office. The Community Space’s mission—guided by patients, caregivers, and the larger healthcare community—supports and encourages patient-centered outcomes through research, education, and community participation. Our vision for the Community Space is that it should be fully integrated into the community through conducting innovative research and education.

Our initial four strategic priorities (as of November 2020) were centered on fall prevention among community-dwelling older individuals. Fall prevention self-care among community-dwelling older adults was identified as a priority area to be addressed in the Community Space based on the Community Space Design Advisory Committee members’ interests and their perceived needs of Galveston County, Texas. In addition, the current research program of the lead faculty (the corresponding author) is likewise focused on fall prevention among community-dwelling older individuals. Through research on aging, the lead faculty’s objective is to embrace holistic health (i.e., caring for the whole person and providing support to meet older adults’ physical, mental, spiritual, or social needs).

The importance of incorporating community engagement in research on fall prevention has been highlighted in the literature [3]. Engaging patients and families in the fall prevention process during acute hospital stays is associated with a 15% reduction in falls and a 34% reduction in fall-related injuries [11,12]. Involving patients, family members, and licensed and unlicensed providers at nursing homes in adopting and implementing an existing fall prevention intervention was also shown to be associated with reducing falls and fall-related injuries [13]. In addition, effective fall prevention interventions should be based on older individuals’ preferences (for example, length of follow-up care), needs, and skills. Clinicians’ relation-building competencies may affect their abilities to put fall prevention knowledge into action with older adults [14,15,16,17,18,19]. Clinicians’ engagement in the intervention-tailoring process and acceptance of strategies are essential [20]. According to a systematic review, the characteristics of older adults who participated in self-care actions to prevent falls included younger males, those who lived with someone, were in self-reported good health, had a fear of falling, demonstrated fall prevention self-efficacy, and were motivated to engage in self-management activities [15]. Research participants’ age and gender are often identified as personal factors influencing self-care behaviors in older adults. However, the majority of the studies included in this review have low strength of evidence [15]. Studies have indicated that prioritizing social connections may reduce a patient’s sedentary behavior while increasing awareness of their physical health and motivating them to participate in fall prevention self-care practices [18,21,22]. When customizing clinical therapies for older adults with and without cognitive impairment, a two-part strategy that includes older adults and their family caregivers should be explored [23,24].

Based on our experience and the recent studies aforementioned, our preliminary strategic priorities in 2020 included the following (details may be requested from the corresponding author by e-mail):

- Infrastructure: Develop a supportive infrastructure that will position the Community Space as a leader in patient-centered outcomes research.
- Training and education: Provide innovative training and education opportunities to enhance the skills and knowledge base of students, faculty, older adults, caregivers, and health care providers such as those in the primary health care and home care settings.
- Research: Promote a viable culture of scholarship using patient-centered outcomes research methods.
- Communication and awareness: Improve fall-prevention knowledge and behaviors among older adults and caregivers through community education and awareness efforts.

### 2.2. Project Implementation through Community Partnership

#### 2.2.1. Learning from Community-Dwelling Older Adults about How to Introduce MedlinePlus and Innovative Devices to Their Peers

The Community Space Design Advisory Committee members started working on grant and project ideas in January 2020. In May 2020, during the COVID-19 pandemic, the UTMB School of Nursing Community Space Design Advisory Committee received the National Network of Libraries of Medicine South Central Region’s Technology Enhancement Award to fund a project titled “Development and Evaluation of a Technology Education for Community and Home Program for Older Adults and Their Family Members and Caregivers to Support Independence (June 2020 to April 2021).” The goal of this initiative was to raise awareness of MedlinePlus (U.S. National Library of Medicine, Bethesda, MD 20894, USA, https://medlineplus.gov/, accessed on 14 July 2021) and market-available devices that improve health and well-being. The proposal of this funded project was co-developed using patient-centered outcomes research methods. The proposal-writing team included five members: one medical reference librarian, two caregivers (who also were health-care providers), one engineer, and one researcher. In addition, this project included three community stakeholders who served community-dwelling older adults in Galveston County, Texas. This effort is associated with multiple priorities of the Community Space (i.e., b. training and education, and d. communication and awareness). Understanding fall prevention self-care is one of the topics discussed in the aforementioned project.

A medical reference librarian (one of the co-authors) shared her personal story and discussed her belief in and commitment to patient-centered outcomes research:

Due to my connective tissue autoimmune disease, I have had many orthopedic surgeries and joint replacements. However, the ability to walk without assistance has never been taken for granted. During my outreach activities, I have been passionate about helping older adults learn how to use medical information and assistive devices to tackle their physical obstacles.

As a medical librarian, I have worked with the first author on multiple outreach projects. For example, I was part of the proposal-writing group for an award from the National Network of Libraries of Medicine that enabled us to supplement funds to the School of Nursing’s Community Space for Health and Well-Being.

During the proposal writing stage, the team sought input from nine community-dwelling older adults. On 3 and 10 February 2020, the team conducted two 90-min volunteer consultation meetings with nine volunteers from the Retired and Senior Volunteer Program (RSVP) at the UTMB Sealy Center on Aging Learning Center. The director of the RSVP at UTMB recruited these volunteers and confirmed their participation via phone for the team. The director of the RSVP and the first author discussed the desire to include participants with diverse ethnic, racial, and economic backgrounds (e.g., males and females, older adults, and family caregivers). Due to privacy concerns, the RSVP director only shared the contact information (i.e., names and phone numbers) of the confirmed volunteers with the first author. The first author was responsible for setting up the meetings and made reminder calls. The consultation meetings were not recorded.

This volunteer group (four men and five women) lived in various parts of Galveston County. Eight of them were over 65 years of age, with one man being 61 years old and actively planning for his future. The volunteers’ ethnic/race groups were as follows: two Hispanic, two Pacific Islanders, two African Americans, and three White. In addition to the volunteers, all five proposal-writing team members and two community partners attended the discussion.

The team met before each meeting and developed the flow and the demonstration plan (i.e., the MedlinePlus website showing selected home technology devices). We used prompts to facilitate the discussions and assigned each team member’s role as the facilitator, presenters, observers, or note-takers. We used PowerPoint and held hands-on experiences (e.g., show and tell in small groups with 1–3 participants). We did not seek informed consent, as the lessons learned from these consultation activities informed grant proposal development. The team also debriefed the lessons learned after each consultation meeting.

The consultation sessions included (1) an introduction to this grant’s aims, including an overview of the proposed course topics; and (2) a tutorial on how to access MedlinePlus, which included a demonstration of the high- and low-technology tools identified for the “Safety and Security” topic. In addition, the volunteers had an opportunity to see, touch, and ask questions about the tools. The research team was able to clarify information and ask additional questions of the volunteers. The volunteers were actively engaged throughout the sessions. Most stayed for 20–30 min after the session’s official ending time to continue sharing ideas and building a network with each other and the researchers. The key lessons learned from these two meetings are summarized below.

At the 3 February 2020 consultation meeting, group members reported that they had access to medical information through the following sources: talking to physicians or primary care providers, reading pamphlets, and searching the internet. Only two members described using the internet to access information. These members reported using the WebMD and Mayo Clinic websites and internet search engines (e.g., Google search engine). None reported using the MedlinePlus website. The initial discussion about high- and low-technology tools focused extensively on tools the members may have heard about but did not use or had not seen (for example, there was considerable interest in understanding an Alexa device [Amazon.com, Inc., Seattle, WA 98109, USA]). Although this first meeting was not conducive to an in-depth demonstration of the technologies, the group provided feedback on the course topics.

Also, during the first meeting, the group agreed that the five class topics were comprehensive while acknowledging that there was some overlap. Note that the proposal writing team members selected the proposed class topics in January 2020. The topics were Stay Active (leisure, exercise, and mobility), Care for Your Body (nutrition and medication), Fear Not (safety and security), Stay Sharp (memory and learning), and Everyday Tools (communications and activities of daily living). The group did not feel the need to add new topics or subcategories to the discussion. The Stay Sharp topic was discussed only briefly. The Fear Not topic sparked a conversation about fall prevention. One team member mentioned tools (for example, dusk-to-dawn light bulbs for keeping the home well lit) which was an interesting new technology to members. The Stay Active topic also included a discussion about fall prevention because a member of the group commented that increasing strength and balance could serve as a way to prevent falling. The Care for Your Body topic was discussed at length by all group members. During a discussion on the subtopic of nutrition, the group expressed considerable interest in cooking classes that focused on high-nutrition/low-cost/convenient recipes using tools such as a microwave, toaster oven, slow cooker, rice cooker, or egg cooker, all of which modify the cooking experience for those who may not use a typical oven or stovetop for cooking meals. Members of the group engaged in creative discussion around the use of low-technology tools, such as an egg slicer that can also be used to slice mushrooms, strawberries, bananas, and other foods; a vegetable chopper that can be used in place of a knife; and a steel chopper that can be used for cutting more significant portions of vegetables without a knife. In a discussion related to medication, the group expressed a lack of awareness of—but interest in—electronic pill reminders that dispense medication at a specific time and blink or buzz until the medication is taken.

At the 10 February 2020 consultation meeting (six members attended), the team picked Topic 3, Fear Not, and used small group discussion (two members per group) to obtain more in-depth insights from the older adults related to (1) their preliminary impression of MedlinePlus, (2) their interest in learning about and potentially adopting high- and low-technology home devices, and (3) the barriers and facilitators to adopting technology that may require wireless technology. Five members said that every home technology tool that was demonstrated—regardless of whether it was high- or low-technology—was new to them. Four members expressed that they wanted to implement at least one of the demonstrated tools. These four members wanted to know where items could be purchased; two of these four members asked the meeting facilitator to write down the names of several items for them. Four of the six members said they have children who could help them set up or monitor the high-technology tools. All members indicated needing some assistance when using the internet to search for health information (for example, to access the MedlinePlus website from their home computer or smartphone).

The implementation team included the one researcher (the first author), one male patient partner, two female caregivers, one medical reference librarian, and one Ph.D. engineer and artist. The team employed patient-centered outcomes research to co-develop and co-implement the patient education course, titled “Innovative Devices to Make Your Life Easier and Happier,” through partnerships with UTMB OLLI, the Moody Library, Health System, and Sealy Center on Aging’s Learning Center. This in-person course, including six face-to-face meetings, was offered at the UTMB OLLI between the period from 27 January to 13 April 2021, and included the topics of MedlinePlus, Stay Active, Care for Your Body, Fear Not, Stay Sharp, and Everyday Tools. The course introduced a Fitbit as an example of a health- and activity-monitoring device and shared a use-case experience about it. The course also included a short lecture about neuroplasticity and brain health.

The implementation team met at least once a week to (1) design the course syllabus (for example, deciding which MedlinePlus topics to include or which devices to demonstrate), (2) solicit input from team members (for example, deciding if the content was appropriate for adults aged 65 years and older), (3) evaluate the feedback from participants and the UTMB OLLI founding director, and (4) develop patient-centered education strategies. The team focused on the knowledge demands of older people and their preferred learning modalities, such as support for using an iPad, the pace of instruction while teaching website navigation, and hands-on practice time. Both MedlinePlus and the course were well-accepted by the participants. The OLLI Founding Director recommended that the team repeat the MedlinePlus portion of the course twice (once in-person and once virtually using Zoom [Zoom Video Communications, Inc., San Jose, CA 95113, USA]) and repeat the portion of the course addressing innovative devices at OLLI in the future.

A community stakeholder partner and the OLLI founding director (one of the co-authors) shared her insights:

A view from OLLI participants’ experiences (related to the National Network of Libraries of Medicine South Central Region, Technology Enhancement Award, titled “Development and Evaluation of a Technology Education for Community and Home Program for Older Adults and Their Family Members and Caregivers to Support Independence”):

Outcomes reported from community members at OLLI demonstrate the interventions’ effectiveness. OLLI participants consistently expressed gratitude for the opportunity to receive sound, evidence-based information from healthcare experts in a relaxed, non-threatening setting. Typical healthcare visits allow insufficient time for detailed information about resources such as the use of MedlinePlus. Popular media offer an overabundance of “health information” with varying amounts of accuracy. Those who participated in patient-centered dissemination activities described here unanimously applauded the quality of content, the knowledgeable presenters, and the ample opportunities for questions and clarifications. The range of UTMB healthcare experts involved in planning and implementation instilled confidence among participants that information was accurate.

From the viewpoint of OLLI participants, the format of the sessions ensured ease of accessibility. Because sessions allowed for interaction among participants and presenters, individual differences, unique health conditions, and lifestyle situations could be fully explored. Because they were dedicated to creating a safe space for learning, presenters motivated participants to experiment with techniques for accessing reliable medical information and utilizing previously unfamiliar devices. As a testament to participants’ appreciation for their experiences, they specifically requested that OLLI repeat patient- and community-based educational experiences, which would allow them and their neighbors to continue these journeys of learning, self-enrichment, and personal empowerment. One participant articulated what represents a consensus: “These highly intelligent healthcare experts communicated effectively at the level laypersons could easily comprehend. Their contagious enthusiasm made learning fun. Even the most basic questions received respectful and thorough answers. Please do more of this!”

#### 2.2.2. Patient-Centered Outcomes Research Training to Support Proposal-Writing Efforts

During the COVID-19 pandemic (2020–2021), a proposal-writing group co-developed three grant proposals for patient-centered outcomes research with local community organizations (for example, the International Nurses Unit of the Church of God in Christ, Inc., Galveston Baptist Association). The composition of the proposal-writing group for each grant proposal varied by grant proposal topics; one group included a team of five members, and the other two groups included a team of 12 members each. Each proposal-writing group engaged at least one patient and two caregivers. The team diligently embraced patient-centered outcomes research methods in proposal development.

A male patient partner (one of the co-authors) shared his personal story and involvement in patient-centered outcomes research:

After a 40-year career, I retired from the Department of Pharmacology & Toxicology at the University of Texas Medical Branch at Galveston as a Professor in 2018. My primary research interests were in the biochemical, cellular, and molecular mechanisms underlying the acute and chronic behavioral effects of drugs of abuse, particularly marijuana cannabinoids, cocaine, and phencyclidine (PCP, “angel dust”).

My primary role in the Community Space for Health and Well-Being is as a “Patient Partner.” Since I am 76 years old and have fallen twice in the last year (while running) and have had occasional short-term memory deficits, I can provide relevant information and “sensibilities” that are not readily available to younger researchers.

I have been actively engaged in the University of Texas Medical Branch at Galveston School of Nursing’s projects and proposal writing that embrace patient-centered outcome research methods. In addition, since July 2020, I have been actively working with researchers, patients, and caregiver partners to identify the needs of the aging population in my community and assist in implementing the Innovative Devices project. I am also interested in learning how to use several innovative “instruments” to help my “well-being.”

The conduct of my research (mentioned above) has required substantial grant request activity, often including revision and resubmission. Hopefully, this experience will help procure the financial means necessary to conduct the fall prevention research of interest by this research group.

In June 2020, the first author recognized that the proposal-writing group, including herself as a researcher, would benefit from learning from experts in patient-centered outcomes research. Therefore, in August 2020, because of COVID-19, the UTMB School of Nursing and Community Space for Health and Well-Being sponsored an online patient-centered outcomes research general training module to be presented to the first author’s proposal-writing group (researchers, patients, caregivers, and clinicians), which included a group of African American faith community nurses. A patient-centered outcomes research consultant provided the 8 h virtual training program.

During this eight hour virtual patient-centered outcomes research training, we learned how to best engage non-researchers in participating at a higher level of health research by discussing the purpose of the research and the basic research concepts and processes with an emphasis on older African Americans. We also identified potential challenges to engaging older African Americans in health research. This training was well-received by the training participants. More details may be requested from the first/corresponding author by e-mail (e.g., accessing to the recorded training).

A community stakeholder, patient and caregiver partner, and faith community nurse in the International Nurses Unit in the Church of God in Christ, Inc. (one of the co-authors) shared this story:

As a faith community nurse and evangelist in the Church of God in Christ, Inc., I have to practice what I preach and teach. My ministry focus is health and healing to a predominantly African American faith-based population and the Galveston County community. In August 2019, at the University of Texas Medical Branch at Galveston School of Nursing, I met the first author, who desired a community advisor who knew Galveston County and the unique needs of its older adult population.

As a former community health nurse, I was well acquainted with both. As a clinician and caregiver partner (caring for my 83-year-old mother, who is walker/wheelchair-bound and prone to fall), I had a unique opportunity to assist with many projects. I assisted in designing the Community Space for Health and Well-Being, participated in several grant-writing efforts, and learned on patient-centered outcomes research and the impact it could have on our community. The International Nurses Unit Faith Community Nurses, the caregivers, the older adult congregants, and other community stakeholders in Galveston County could embrace outcomes through research and education.

The International Nurses Unit Faith Community Nurses partnered with UTMB to build our health ministries and to edify the church and our faith-based community. We could plan our health promotion activities with our members and assess the outcome of training and research. To sustain the patient-centered outcome research efforts, our faith community nurses, who were trained in research, will be capable of training faith community nurses to become better health promotion researchers by using virtual and in-person opportunities.

The patient-centered outcomes research training is very informative, and I learned from other team members and the African American nurses. African American older adults need to feel comfortable talking to researchers. We need to empower our African American older adults to engage in mutual dialogues that foster meaningful input. If African Americans have questions, they will be comfortable sharing their concerns with us.

Our Bible scripture, which fuels our goal for the people of God, says that we should strive “to prosper and be in health even as thy soul prosper.” (3 John:2).

We got a chance to practice what we preached! We wanted the African American older adults to become accustomed to research and education, overcome mistrust of the medical community, and allow African American voices to be heard. We also believe we can reach members of the faith-based community and encourage them to promote health research to others in the Church of God in Christ, Inc.

A female caregiver of her mother-in-law and a nurse of the Catholic faith (one of the co-authors) shared her experience with the patient-centered outcomes research training provided by the Community Space for Health and Well-Being:

I have been a family caregiver for my mother-in-law, who lives with me, and a virtual family caregiver for my mother, who lives in southern Asia with one of my sisters, for over 20 years. My mother-in-law is a Muslim. Understanding her belief and cultural practice related to self-care, such as preventing falls at home, is important for me to be an effective family caregiver. As a nurse for more than 30 years, I have been passionate about helping older adults stay independent and stay in their own homes as long as they are able and want to.

The August 2020 patient-centered outcomes research training was very informative, and I learned a lot. I believe that researchers, clinicians, patients, caregivers, and stakeholders need to have shared knowledge about patient-centered outcomes research. We must collaborate with our patient partners, especially older adults, on developing solutions that will meet their learning styles and help them perform their activities of daily living. We need to hear their voices and learn about their values through listening, focusing on their beliefs and preferences, and soliciting active participation, thereby building trust.

## 3. Conclusions and Practical Implications

As with the Meharry Medical College Community Engagement Core development [5], the UTMB School of Nursing took a similar path to cultivate community-academic research partnerships and embrace patient-centered outcomes research. One significant difference is that the Meharry Medical College Community Engagement Core received funding support from the National Institutes of Health/National Institute of Minority Health and Health Disparities. Both the Meharry Medical College Community Engagement Core and UTMB School of Nursing took the following steps to intentionally engage community stakeholders in all research phases:

(a) Developed the mission and vision statements with communities.
(b) Established a community advisory board to build trust and provide opportunities for community members to have representation in research activities.
(c) Provided training and workshops to community members.
(d) Promoted research relevance among community stakeholders.
(e) Continuously sought community stakeholder feedback on research or project procedures.

For academic nurses to practice what they preach about patient-centered outcomes research, a “one-size-fits-all” technique is no longer an option. For example, fall prevention programs should be tailored to older adults and their family caregivers’ unique needs across the care continuum in settings with diverse environmental resources (for example, in urban areas with easily accessible transportation, which allows for more social involvement). In addition, the process of engaging patients and their family caregivers in fall prevention self-care should be simple (for instance, linking hospital and community efforts) [21,23]. Researchers and clinicians could not achieve such outcomes without using patient-centered outcomes research to solicit the inputs of the end-users (older adults, their caregivers, licensed and unlicensed nurses, and other healthcare team members) [20].

Whether academic nurses are practicing what they preach in terms of patient-centered outcomes research remains uncertain. More examples from academia are required to make that determination. In this work, we provided a real-world example to demonstrate practicality. Academic nurses are embarking on a steep learning curve to integrate patient-centered outcomes research into scholarly efforts. Their journey will necessitate the engagement of a team consisting of (but not limited to) researchers, doctors, patient and caregiver partners, and other stakeholder partners. Such a community engagement research journey requires patience and a systematic plan with needs ranging from an academic, enterprise-wide infrastructure to resource distribution at a local level. Thus, for the educational settings that do not have an existing infrastructure to support patient-centered outcomes research, researchers may encounter challenges related to issues such as training needs (for instance, training for researchers and community members to engage in patient-centered outcomes research) and allocated funding, researcher and staff efforts, and time [5].It would be helpful to adopt existing national resources for patient-centered outcomes research training through the Patient-Centered Outcomes Research Institute in the United States. The Patient-Centered Outcomes Research Institute offers free comprehensive training materials for researchers and non-researchers to learn about the health research process and how to become involved in patient-centered outcomes research in their publication titled “Research Fundamentals: Preparing You to Successfully Contribute to Research [25].” Such training would be essential to establish a meaningful partnership between academia and communities and develop shared research goals and objectives [5]. 

In summary, we have shared our journey on how we may start adopting an experiential exercise or design thinking process to understand and address human needs to promote patient safety (i.e., supporting older adult independence and respecting their preferences to stay in their own homes as long as they are able and want to) [26]. Our journey included a collaborative partnership with older adult patients and their caregivers to develop solutions to meet patients’ learning styles and help them perform their activities of daily living. In the idea development and implementation phases, we put older adult patients at the center of the project scope. We intended first to seek to understand the essential needs and considerations for a successful intervention. We believe that patient-centered outcomes research methods could minimize challenges arising from perfect solutions that become unadaptable because of invisible barriers that researchers fail to discover during the initial design process [27,28,29]. We valued engaging stakeholders in the design and delivery of the solution/intervention (e.g., workflow, approaches for success) and dissemination of study results (e.g., manuscript writing and presentations) [27,28,29].

The MakerHealth Space’s engineer (one of the co-authors) at the UTMB Health System Department of Nursing Science Innovation shared her perspective about the design of the Community Space for Health and Well-Being:

The Community Space for Health and Well-Being design and its activities have been collaborative efforts encompassing a diverse and interdisciplinary approach. Each member brings their expertise to contribute to a well-rounded, relevant, and well-informed effort. In collaborating with the MakerHealth Space, we have incorporated tools and techniques from a design-focused perspective, allowing for patient-centric interventions to be created with human-centered design principles in mind.

The design-focused methodology approaches problem-solving by observing human behavior, directly interviewing end-users to learn what matters to them, and placing those concerns at the top to arrive at the best possible integrated solutions [26]. This human-centered design puts people at the center of the scope and reveals the essential needs that should be met for a successful intervention. Although other approaches focus on problems or solutions, this method re-centers the objectives. It shows on its own the necessary and vital aspects that will make an intervention work. Patient-centered outcomes research methods could alleviate pain points arising from perfect solutions that become unadaptable because of invisible barriers that were not discovered during the initial design process.

Discipline diversity gives rise to multiple perspectives on problem-solving, but what is always consistent is that interventions should be based on evidence and data, tested, and repeated, as iteration is the key to excellence and improvement.

## Figures and Tables

**Table 1:** Summary of the related activities by dates at the University of Texas Medical Branch (UTMB) at Galveston School of Nursing.

Date	Activities
2019–2025	The UTMB School of Nursing developed a strategic plan titled “Vision 2025,” which emphasized the importance of engagement by expanding professional networks and community interactions to generate opportunities for students, faculty, and alumni to promote long-term beneficial collaborations [6]. The UTMB School of Nursing has begun implementing a systematic approach to support patient-centered outcomes research.
2020–2021	The UTMB School of Nursing’s Research Innovation and Scientific Excellence (RISE) Center developed its strategic plan [7], which emphasizes the importance of community engagement and interdisciplinary collaborations.
August 2019–January 2021	The first author received the University of Texas System Rising Science and Technology Acquisition and Retention (STARs) Award for equipment, repair, and renovations funded from Permanent University Fund bond proceeds in August 2019. These funds were used to renovate the space to be used for a new Community Space for Health and Well-Being (henceforth referred to as the Community Space) within the UTMB School of Nursing’s RISE Center. The refurbishment of this facility was completed in January 2021.
October 2019	The Community Space Design Advisory Committee was formed. The committee members have been actively engaged in grant proposal development since the committee was formed.
September–November 2020	The Community Space Design Advisory Committee engaged in the development of the mission, vision, and value statements. The preliminary versions of the mission, vision, and value statements were developed on the basis of feedback from the Community Space Design Advisory Committee beginning in the fall of 2020 through meetings, surveys, and interviews with five patients or caregivers.
May 2020–April 2021	In May 2020, during the COVID-19 pandemic, the UTMB School of Nursing Community Space Design Advisory Committee received the National Network of Libraries of Medicine South Central Region’s Technology Enhancement Award to fund a project titled “Development and Evaluation of a Technology Education for Community and Home Program for Older Adults and Their Family Members and Caregivers to Support Independence.” Note that the National Network of Libraries of Medicine is now the Network of the National Library of Medicine.

## Data Availability

Not applicable.
